# Editorial Peer Reviewers' Recommendations at a General Medical Journal: Are They Reliable and Do Editors Care?

**DOI:** 10.1371/journal.pone.0010072

**Published:** 2010-04-08

**Authors:** Richard L. Kravitz, Peter Franks, Mitchell D. Feldman, Martha Gerrity, Cindy Byrne, William M. Tierney

**Affiliations:** 1 Department of Medicine, University of California Davis, Sacramento, California, United States of America; 2 Department of Family and Community Medicine, University of California Davis, Sacramento, California, United States of America; 3 Department of Medicine, University of California San Francisco, San Francisco, California, United States of America; 4 Department of Medicine, Oregon Health & Science University Portland, Oregon, United States of America; 5 Regenstrief Institute, Indianapolis, Indiana, United States of America; Children's Hospital of Eastern Ontario, Canada

## Abstract

**Background:**

Editorial peer review is universally used but little studied. We examined the relationship between external reviewers' recommendations and the editorial outcome of manuscripts undergoing external peer-review at the *Journal of General Internal Medicine* (JGIM).

**Methodology/Principal Findings:**

We examined reviewer recommendations and editors' decisions at JGIM between 2004 and 2008. For manuscripts undergoing peer review, we calculated chance-corrected agreement among reviewers on recommendations to reject versus accept or revise. Using mixed effects logistic regression models, we estimated intra-class correlation coefficients (ICC) at the reviewer and manuscript level. Finally, we examined the probability of rejection in relation to reviewer agreement and disagreement. The 2264 manuscripts sent for external review during the study period received 5881 reviews provided by 2916 reviewers; 28% of reviews recommended rejection. Chance corrected agreement (kappa statistic) on rejection among reviewers was 0.11 (p<.01). In mixed effects models adjusting for study year and manuscript type, the reviewer-level ICC was 0.23 (95% confidence interval [CI], 0.19–0.29) and the manuscript-level ICC was 0.17 (95% CI, 0.12–0.22). The editors' overall rejection rate was 48%: 88% when all reviewers for a manuscript agreed on rejection (7% of manuscripts) and 20% when all reviewers agreed that the manuscript should *not* be rejected (48% of manuscripts) (p<0.01).

**Conclusions/Significance:**

Reviewers at JGIM agreed on recommendations to reject vs. accept/revise at levels barely beyond chance, yet editors placed considerable weight on reviewers' recommendations. Efforts are needed to improve the reliability of the peer-review process while helping editors understand the limitations of reviewers' recommendations.

## Introduction

Editorial peer review is widely regarded as the cornerstone of quality assurance in academic medical scholarship.[1], [2] Aside from its broader purpose of fomenting scientific discourse, peer review serves two instrumental functions: to improve quality of research reporting (“quality improvement”) and to aid editors in deciding whether to accept submitted work (“filtering”). The *Journal of General Internal Medicine* (JGIM) is a peer-reviewed journal focusing on clinical care, education, and research in general internal medicine and primary care. Like many of their peers, *JGIM* editors claim that “the quality of the papers published in the *Journal* depends on both the authors and the external reviewers who help the editors select the best papers and improve their presentation.”[3]

We distinguish here between reviewer *recommendations* (i.e. accept/revise vs. reject) and reviewer *comments* (narrative assessment and suggestions for improvement). To the extent that JGIM editors are influenced in their decisions by reviewer recommendations, “it is reasonable to expect that experts' judgment[s] be somewhat concordant.” [4]

Do peer reviewers assigned the same manuscript tend to issue similar judgments? Data are conflicting. One early study from the *Journal of Clinical Anesthesia* found moderate levels of reviewer concordance (40% of papers received identical recommendations from two reviewers and an additional 40% differed by only one category).[5] However, published data from the fields of radiology[6], clinical neuroscience[7], and rehabilitation[8] suggest that chance-corrected agreement between reviewers is only fair. Aside from studies of abstracts submitted to scientific meetings,[9]–[12] there are to our knowledge only two, relatively small, studies of the issue in non-specialty journals, conducted in Croatia[13] and India[14]. In addition, since publication of Siegelman's classic contribution[15], few analyses have addressed the relative contribution of article quality (hypothetically, an intrinsic property of the manuscript) vs. reviewer style (possibly ranging from highly skeptical to relatively uncritical) in generating recommendations to publish or reject. A disproportionate contribution of reviewer style would raise additional questions about current mechanisms of peer review.

While perfect agreement among reviewers is arguably unnecessary (implying redundancy of effort) or undesirable (perhaps suggesting excessive cognitive homogeneity in the reviewer pool), editors should expect reviewer recommendations to be substantially more consistent than mere chance. How much more consistent? The answer may depend on a particular journal's target acceptance rate and decision structure. Hargens suggests that editors of more selective journals put greater credence in negative reviews, whereas editors of less selective journals assign greater weight to positive reviews. These editorial predispositions may influence who is asked to review, how many reviews are requested, and how discordant recommendations are reconciled.[16] However, even allowing a healthy degree of variation in journal policies and practices, it would seem reasonable to expect that inter-reviewer consistency exceed chance by at least 20% (i.e., kappa > = 0.20, commonly viewed as only fair agreement beyond chance).[17]

We conducted the current study to address three questions. First, to what extent do peer reviewers at JGIM agree with each other in their manuscript recommendations? Poor agreement would suggest that reviewer *recommendations* (though not their *comments*) are not meaningful and should therefore be ignored – or at least steeply discounted – in making editorial decisions. Second, to what extent do JGIM editors incorporate reviewer recommendations into their decisions? For example, do they place more weight on reviewer recommendations than the data warrant? Finally, to what extent does reviewer style influence recommendations? The results have implications for the editorial process, and in particular how journal editors should collect, assess, and act on reviewer recommendations.

## Methods

### Data collection and management

Information on all 6213 manuscripts received by JGIM between 2004 and 2008 (inclusive) were stored in a central database at the Regenstrief Institute (Indianapolis, Indiana). Each submitted manuscript underwent two levels of initial internal editorial screening. First, one of the Co-Editors-in-Chief read the abstract. Articles felt to be inconsistent with the journal's mission were rejected. The remainder were assigned to a Deputy Editor with expertise relevant to the content of the article. The Deputy Editor then decided whether to reject the article without external review or to send the article out for external peer-review. JGIM routinely sought three peer-reviewers for each manuscript. We analyzed results for the 2264 manuscripts (36%) that were sent out for external review. Sources of data included structured forms completed by peer reviewers (1–4 per manuscript) and final editorial decisions made by JGIM's Editors (including Editors-in-Chief and Deputy Editors)) (1 per manuscript). Neither the Co-Editors-in-Chief nor Deputy Editors were blinded to the manuscripts' authors or institutions. For the first three years of this study, reviewers were not provided with manuscript authors or institutions, although no other efforts were made to blind the reviewers (e.g., removal of references to the authors' prior publications). In the fourth year of this study, the authors and their institutions were provided to the reviewers to give reviewers the opportunity to comment on possible conflicts of interest.

### Data analysis

Data were analyzed using Stata (version 11.0, StataCorp, College Station, TX). We used kappa statistics to evaluate chance-corrected agreement among reviewers' recommendations to reject vs. accept/revise specific manuscripts. We used mixed model logistic regression analyses, with individual reviewer recommendation as the unit of analysis, to adjust reviewers' recommendations for review year and manuscript type (original research vs. other). We used random effects models to account for nesting of reviews by manuscript and by reviewer and to calculate the intra-cluster correlation coefficients (ICCs) for manuscripts and reviewers. Because of the cross-nested nature of reviewers and manuscripts, simultaneous consideration of these variables as crossed random effects within the same logistic regression using the entire dataset exceeded the available computing capacity. Therefore, the reported ICCs are based on considering manuscripts and reviewers as the random effect in separate analyses. The results were consistent with those based on two other models using a 10% random sample of the data; each model treated one of the variables as a fixed effect and the other variable, in turn, as a random effect. We conducted supplementary analyses to examine possible Deputy Editor effects. (At JGIM, Deputy Editors have delegated authority to accept or reject manuscripts.) In these supplementary analyses, the unit of analysis was the manuscript, the dependent variable was the Deputy Editor decision (reject vs. not reject), and Deputy Editor was treated as a random effect.

## Results

### Editorial outcomes of submitted manuscripts

**Figure 1:**
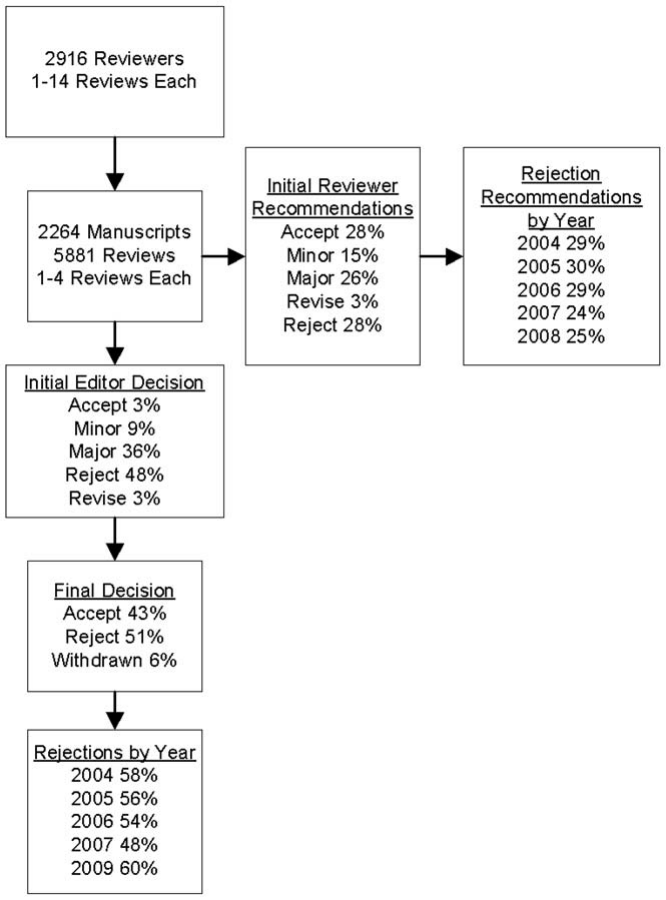
Flow chart showing outcome of reviews pertaining to 2264 manuscripts undergoing external peer review at the *Journal of General Internal Medicine*.

The 2264 manuscripts sent for external review during the study period received 5881 reviews provided by 2916 reviewers; 3.5% received one review, 34.6% received two reviews, 60.6% received three reviews, and 1% received four reviews. (Figure 1). Each reviewer conducted an average of 2.9 reviews during the study period (median 2, range 1–14). Among all reviews, 28% recommended rejection, 28% recommended acceptance (8% unconditional, 20% conditional), and 45% recommended revisions (15% minor, 26% major, 3% unspecified). Among the 2264 manuscripts, 43% were ultimately accepted, 51% were rejected, and 6% were withdrawn (Figure 1).

### Reviewer agreement

The kappa statistic for inter-reviewer agreement on reject vs. accept/revise for each manuscript was 0.11 (p<.001); it was 0.14 when there were 4 reviews, 0.12 when there were 3 reviews, and 0.08 when there were 2 reviews. In a mixed effects logistic regression (taking the reviewer's recommendation to reject as the outcome; manuscript type and year of submission as fixed effects; and manuscript identity as a random effect) the rho coefficient (ICC) for manuscript identity was 0.17 (95% CI 0.13–0.22), confirming modest inter-reviewer agreement. The mixed effects model using reviewer identity as the random effect yielded a reviewer ICC of 0.23 (95% CI, 0.18 to 0.29, data not shown in tabular form.) Assuming an ICC of 0.17 (i.e. the manuscript-level ICC observed in the current study), 7 reviewers would be required to achieve a Cronbach's alpha reliability of 0.6 and 18 reviewers would be needed to yield an alpha of 0.8. With a 50% increase in the manuscript-level ICC, 4 reviewers would suffice for an alpha of 0.6 and 10 for an alpha of 0.8. With a 100% increase in ICC, the requisite number of required reviewers would be 2 (for an alpha of 0.6) and 6 (for an alpha of 0.8).

### Editorial decision-making in relation to reviewer recommendations

Among the 2264 manuscripts reviewed during the study period, just under half received reviews that were in complete agreement *not* to reject (i.e., *all* reviewers recommended accept/revise), less than 10% received reviews that were in complete agreement to *reject*, and the balance received reviews with conflicting recommendations (Table 1). The editors rejected 48% of 2264 manuscripts sent out for external peer-review. If all reviewers recommended *not* to reject, editors rejected the manuscript 20% of the time. If all reviewers recommended “reject,” editors rejected 88% of the time. And if reviewers were divided, editors rejected the manuscript 70% of the time (p<.001, Table 1). There was no significant relationship between the number of reviews and the initial editorial decision to reject (chi-square = 1.9, degrees of freedom = 3, p = 0.60).

**Table 1:** Likelihood of Initial Decision to Reject in Relation to Reviewer Agreement.

Reviewer Recommendations	N (%)	Fraction Rejected by Editors (%)
Complete agreement *not* to reject	1080 (47.7)	20.3
Any level of disagreement	1027 (45.4)	70.6
Complete agreement to reject	157 (6.9)	88.5
Total	2264 (100)	47.8

### Deputy editor effects

The 57 Deputy Editors managed 2–179 manuscripts (mean 102). In an analysis using manuscript as the unit of analysis and the assigned Deputy Editor as a random effect, taking initial editorial decision (reject or not) as the dependent variable, and no independent variables, the Deputy Editor ICC was 0.02 (95% CI, 0.01 to 0.06). When this model adjusted also for reviewer agreement (complete agreement to reject, complete agreement to accept, or disagreement), manuscript year and article type, the Deputy Editor ICC increased to 0.03 (95% CI, 0.02 to 0.08).

## Discussion

The results of this analysis suggest that reviewers for JGIM agreed on the disposition of manuscripts at a rate barely exceeding what would be expected by chance. Nevertheless, JGIM editor's decisions appeared to be significantly influenced by reviewer recommendations. In particular, agreement by all reviewers that a manuscript should be rejected (an uncommon occurrence in our data) essentially sealed its fate. Consensus among reviewers that a manuscript deserved further consideration (either an opportunity to revise and resubmit or conditional acceptance) reduced the likelihood of rejection from approximately half (for all manuscripts sent out for peer-review) to about one in five. These results challenge biomedical journal editors to reconsider what is now standard practice: asking reviewers to provide recommendations to accept, revise, or reject submitted manuscripts.

Of the two instrumental purposes served by peer review (quality improvement, and decision making or “filtering”), filtering is arguably less important to the biomedical enterprise as a whole, since most rejected manuscripts eventually get published. [18], [19] In addition, some journals have explicitly rejected the filtering function, promising to publish all manuscripts within the journal's scope that are “technically sound” (http://www.plosone.org/static/information.action). For the time being, however, placement of a manuscript within a particular journal is of great significance to readers, authors, and authors' institutions. Busy clinician-readers fix their attention on a limited number of journals, chosen according to the journals' clinical focus and impact.[20], [21] Authors expend considerable energy preparing articles for specific journals, slanting their presentation to meet the needs and expectations of particular readerships. Academic institutions use publication venue as a factor in making tenure and promotion decisions.[22] As long as journals are complicit in this process, they have a duty to assess and improve the processes they use to accept or reject articles for publication.

In this light, the results of this study are provocative. Reliability is a pre-requisite for validity, and the reliability of reviewer recommendations at JGIM (and possibly at other journals) is low. Peer review serves multiple purposes, including social functions such as managing the process by which a “community certifies additions to its body of accepted knowledge”[16]. In addition, editors may put reviewers' recommendations to some less-than-obvious purposes. For example, some editors may use the recommendations “check box” to calibrate reviewers' narrative comments, especially those visible to authors. Nevertheless, we are unpersuaded that because “reviewers advise and editors decide,” inter-reviewer reliability is moot. Like JGIM, most biomedical journals routinely ask reviewers for summative advice about priority for publication. If reviewers cannot regularly agree on whether to recommend rejection or further consideration, the marginal contribution of such summative recommendations may be small, and worse, they may distract from reviewers' primary contribution, which is to improve the reporting – and ultimately the performance – of science.

Several solutions might be entertained. First, editors might solicit more reviewers per manuscript; however, given ICCs in the range reported here, it would take 18 reviews per manuscript to push the coefficient alpha above 0.8. Given the difficulty JGIM[23] and other journals are having securing peer-reviews, such a recommendation is impractical. However, it might be applicable to post-publication review, using an approach like that of the McMaster Online Rating of Evidence program.[24]

A second solution is to improve the process of peer review by providing more effective guidance to reviewers or by working to enhance the psychometric properties of the questions that are posed to them. JGIM hosts an annual peer review workshop at a national meeting but (for obvious reasons) does not *require* attendance. JGIM also provides guidelines for reviewers on its website (http://jgim.iusm.iu.edu/) but does not monitor website traffic nor formally certify reviewer competence. More formal attempts at reviewer training have met with mixed results.[25] With regard to measurement, most journals ask reviewers to rate different dimensions of manuscript quality on makeshift Likert scales. However, the reliability and validity of these scales and their relation to reviewer recommendations require further testing.[26]

Finally, journal editors could consider a break with tradition by dispensing with reviewer recommendations altogether, asking them to focus instead on evaluating the strengths and weaknesses of manuscripts across multiple dimensions and particularly on suggestions for improvement. Under this approach, the role of the reviewer would be realigned to emphasize evaluation and constructive criticism rather than decision-making. Again, such an approach should undergo a formal evaluation.

It is interesting to note that recommendations were more consistent for multiple manuscripts assigned to the same reviewer (intra-class correlation coefficient rho = 0.23) than for multiple reviewers assessing the same manuscript (rho = 0.17). These results provide evidence that reviewers have an evaluation style that exerts itself across manuscripts. Any college undergraduate knows that there are hard and easy graders among professors, but it is still surprising that the propensity of reviewers to be generous or tough is quantitatively larger than the tendency of different raters to recommend rejection (vs. further consideration) of the same manuscript. In a study conducted at the American Journal of Radiology, Siegelman identified 8 “zealots” (very easy graders) and 9 “assassins” (very hard ones) among 660 reviewers who had reviewed at least 10 manuscripts. Our data indicate that inter-reviewer variability is much more pervasive; it is not just outliers who adopt a particular “style.” The existence of such a style effect and its potential arbitrary influence on the fate of manuscripts also raises questions about the probative value of reviewer recommendations.

In contrast to the evidence for a modest reviewer style effect, there was little evidence for a substantive Deputy Editor style effect on initial rejection decisions. While the Deputy Editor rho was statistically significant, it was small (0.02) and increased only to 0.03 after adjusting for the effect of reviewer agreement, manuscript year and article type. It remains possible that editors exert a style effect through their selection of specific reviewers.

This report has certain limitations. Most importantly, the data were obtained from a single general medical journal, and generalizability is therefore limited. However, manuscripts submitted to JGIM encompass a wide array of topics including clinical and health services research, clinical medicine, medical education, and health policy, so these findings may be relevant to a wide array of general medical journals. Moreover, many JGIM reviewers are academic general internists with extensive training in clinical epidemiology, health services research, critical appraisal, and biostatistics. Concordance within this methodologically minded cohort of reviewers might be expected to be, if anything, higher than for the average clinical journal. Other limitations include the relatively short evaluation period, likely non-random assignment of manuscripts to reviewers (editors may choose “hard graders” to review papers they don't like), and lack of data on outcomes (e.g., citation counts for articles published with reviewer concordance vs. those published despite reviewer disagreement). Further, we did not investigate the informational content of reviewers' narrative comments nor their impact on editorial decision-making. It remains possible that such comments drive editorial decisions in a more reliable and valid fashion than reviewers' summary recommendations. Finally, there is no guarantee that any of the potential solutions discussed here will work better than the current system. A Cochrane review concluded there is scant evidence to support the current process of editorial peer review.[27] Further study, including randomized controlled trials, is needed before implementing changes to a system that has withstood the test of time, if not scientific scrutiny.

In summary, reviewer publication recommendations over a five-year period at the *Journal of General Internal Medicine* showed scant agreement but were nonetheless accorded considerable weight by the editors. Reviewers appear to have a relatively stable style that influences their recommendations over time and across manuscripts. Biomedical journal editors should look carefully at their own data – and pool data across journals – in an effort to create a more reliable and valid review process.
